# Gender Differences and Postoperative Delirium in Adult Patients Undergoing Cardiac Valve Surgery

**DOI:** 10.3389/fcvm.2021.751421

**Published:** 2021-11-23

**Authors:** Hongbai Wang, Xiaoxiao Guo, Xianlin Zhu, Yinan Li, Yuan Jia, Zhe Zhang, Su Yuan, Fuxia Yan

**Affiliations:** ^1^Department of Anesthesiology, Fuwai Hospital, Peking Union Medical College, Chinese Academy of Medical Sciences, Beijing, China; ^2^Department of Cardiology, Peking Union Medical College Hospital, Chinese Academy of Medical Sciences, Beijing, China; ^3^Department of Anesthesiology, The Central Hospital of Enshi Tujia and Miao Autonomous Prefecture, Enshi City, China

**Keywords:** gender, cardiac surgery, postoperative delirium, adult, risk factor

## Abstract

**Background:** Postoperative delirium (POD) is common in patients following cardiac surgery. According to studies on non-cardiac surgery, males suffered from higher incidence of POD. However, there is no report about effect of gender differences on POD occurrence in cardiac surgery patients. The aim of this study was to investigate the effect of gender differences on POD occurrence in adult patients after cardiac valve surgery.

**Methods:** This is a retrospective case-control study. We recorded the clinical data in adult patients who underwent elective cardiac valve surgery from May 2019 to October 2020. POD was assessed by the Confusion Assessment Method for Intensive Care Unit. Univariate analysis was used to screen the potential risk factors. Collinearity analysis was conducted to detect overlapping predictor variables on the outcomes. A multivariate logistic regression with odds ratio (OR) and 95% confidence interval (CI) was used to identify the independent risk factors. The Hosmer-Lemeshow test was performed to show the good calibration of the logistic regression model.

**Results:** In total, we recorded the perioperative data in 431 adult patients, including 212 males and 219 females. Sixty patients suffered from POD, including 39 males and 21 females. Twenty-one perioperative variables were selected, and 11 were screened by univariate analysis. We did not detect the severe collinearity among the 11 variables. Male gender was identified as a significant risk factor in POD occurrence in patients undergoing cardiac surgery (Adjusted OR: 2.213, 95% CI: 1.049–4.670, *P* = 0.037). The Hosmer-Lemeshow test demonstrated good calibration of the logistic regression model (χ^2^ = 7.238, *P* = 0.511). Besides, compared with females, the relationship of male and delirium subtypes was as follows: (1) hyperactive: adjusted OR: 3.384, 95% CI: 1.335–8.580, *P* = 0.010; (2) hypoactive: adjusted OR: 0.509, 95% CI: 0.147–1.766, *P* = 0.287. A Stratification analysis by age demonstrated that the males showed higher POD incidence in patients aged younger than 60 years (adjusted OR: 4.384, 95% CI: 1.318–14.586, *P* = 0.016).

**Conclusions:** Male gender is an important risk factor in POD occurrence in patients following cardiac surgery. Furthermore, the incidence of hyperactive delirium is higher in males. Besides, the male patients aged younger than 60 years are at high risk of POD. We should pay more attention to the male patients to prevent their POD occurrence.

## Introduction

Postoperative delirium (POD) is a series of mental disorders characterized by acute and paroxysmal onset following surgery. The distinctive clinical manifestations of POD include inattention, disorganized thinking and altered states of consciousness (1). POD commonly occurs in patients undergoing major cardiovascular or major non-cardiac surgery during the first 3 days postoperatively (2, 3). However, relative to non-cardiac surgery, the incidence of POD in patients with cardiac surgery was even higher, and accounted for 5–72% (4, 5). Delirium is significantly associated with an elevated incidence of long-term cognitive dysfunction, compromised life quality and even mortality (6–10). Given that the specific mechanism of POD is complex and still unclear, thus, there is no effective method to cure it, once POD occurs. Currently, the best way to reduce the POD incidence is to manage its risk factors (11).

So far, some risk factors of POD have been identified, such as advanced age, perioperative poor sleep quality, preoperative cognitive dysfunction, perioperative use of hypnotics and sedatives, postoperative hyperalgesia, postoperative long-time mechanical ventilation, and so on (12). A multicenter study demonstrated that males had a higher scores on motor agitation than females in patients with delirium (13). Additionally, male patients suffered from higher POD incidence in older patients (age ≥ 65 years) undergoing hip fracture surgery (14). But there is no report about the association between gender differences and POD incidence in cardiac surgery patients. This study was designed to investigate the effect of gender differences on POD occurrence in patients undergoing cardiac valve surgery.

## Methods

This was a retrospective case-control study. The Ethics Committee of the Chinese Academy of Medical Sciences Fuwai Hospital in Beijing approved this study (Approved number: 2020-1330). We continuously selected the adult patients (age ≥18 years) with ASA grading II-III, who underwent cardiac valve surgery under cardiopulmonary bypass (CPB) from May 2019 to October 2020 in our center. The exclusion criteria were: (1) patients having a history of delirium, dementia, epilepsy, or schizophrenia; (2) patients having a history of brain trauma or brain surgery; (3) patients with left ventricular ejection fraction (LVEF) ≤ 30%; (4) patients having severe preoperative liver (Child–Pugh grading C) and renal (stage 3–4 according to Chronic Kidney Disease grading) dysfunction; (5) patients who did not provide the POD information because of death or no response to any stimulates during the follow–up period; (6) patients suffering from re-operation during the follow–up period.

### Anesthesia Programme

All included patients received general anesthesia of the same anesthesia programme. The induction medications of general anesthesia were midazolam, sufentanil, etomidate, and cisatracurium. Anesthetics sustaining anesthesia included: propofol, sufentanil, cisatracurium, and sevoflurane. We used the Bispectral Index (BIS) to monitor the depth of anesthesia, and maintained BIS values between 40 and 60 during surgery. A normal body temperature was maintained except for light hypothermia (nasopharyngeal temperature: 30–34°C) during aortic clamping.

### Assessment of Preoperative Cognition and Sleep Quality

We evaluated the preoperative cognitive state of participants through the Mini-Mental State Examination (MMSE) which scores from 0 to 30 (15). The Pittsburgh Sleep Quality Index (PSQI) is an effective method to assess preoperative sleep quality during the recent month. It includes 18 items in seven parts, and its score ranges from 0 to 21 (Supplementary Material 1). A value of five in the PSQI is considered as a cutoff point of poor and good sleep quality for adults. The higher the PSQI score is, the more serious the poor sleep quality is (16).

### POD Assessment

The medical staff strictly trained by a senior psychologist assessed POD of enrolled patients. The Confusion Assessment Method for Intensive Care Unit (CAM–ICU) is commonly used in delirium assessment, which contains four parts: (1) acute change of mental status and behavior during the past 24 h; (2) inattention; (3) disordered thinking and (4) altered state of consciousness (Supplementary Material 2). And its simplified Chinese version has been developed and is a valid assessment tool in Chinese population (17). POD assessment consists of two main processes. First, follow-up staff assessed the sedation state of included patients by the Richmond Agitation-Sedation Scale (RASS) (Supplementary Material 3) (18). Only when the RASS score was −3 or above, was the assessment of delirium processed through the simplified Chinese version of CAM–ICU (17). The diagnosis of delirium is identified when items (1), (2) and (3) or (4) are met (19). Besides, delirium can be divided into three types according to RASS scores: (1) hypoactive type: RASS score is <0; (2) hyperactive type: RASS score is more than 0 and (3) mixed: alternate occurrence of hypoactive and hyperactive types (20). The delirium was assessed only once in the first postoperative 24 h, and the time of assessment was as close as possible to 24 h of the end of the surgery to minimize the impact of anesthetics on the consciousness of patients (21). From the second to the 50 days postoperatively, delirium was assessed twice daily (8:00–10:00 and 18:00–20:00).

### Perioperative Parameters

We selected 21 perioperative demographic data or potential risk factors based on the previous studies and clinical experience (22, 23). Two researchers independently collected the perioperative parameters based on medical record. These perioperative data included age, gender, body mass index (BMI), educational level, smoking, alcohol abuse, preoperative MMSE scores, preoperative sleep quality, history of cerebrovascular disease, history of diabetes mellitus (DM), anesthesia duration, surgery duration, CPB duration, aortic clamping and hypothermia duration, intraoperative blood loss, intraoperative infusion volume, post-surgery mechanical ventilation time, ICU stay time, the postoperative highest numerical rating scale for pain (NRS), postoperative sleep quality, and the number of patients with POD. A third researcher reviewed the accuracy of the data.

### The Study Endpoint

The endpoint of this retrospective study was the association of gender on POD incidence during the first 5 days following cardiac surgery.

### Statistical Analyses

SPSS 25.0 (IBM Corp., Armonk, NY, USA) was used to analyze the data. Continuous variables were presented as median and interquartile range (IQR). Categorical variables were expressed as numbers and percentages (%). The method of multiple imputation was used to manage data loss. Univariate analyses: continuous variables were analyzed through Mann–Whitney *U*-test, and categorical variables through Pearson's Chi-square test. A binary logistic regression model with odds ratio (OR) and 95% confidence interval (CI) was used to analyze the correlation of gender and other covariates that were found to have a *P*-value < 0.1 in the univariate analysis. Model-fitting of the logistic regression models were assessed using the Hosmer-Lemeshow goodness of fit test. The collinearity analysis to detect the overlapping predictor variables on the outcomes, and the variance inflation factor (VIF) >10 or tolerance <0.1 was a criteria of severe collinearity (24). A *post-hoc* analysis based on delirium subtypes was performed to observe the association between gender and delirium through logistic regression model. The statistical significance was marked by a *P-*value of < 0.05.

### Sample Size Calculation

PASS 15.0 (NCSS, Kaysville, UT) software was used to evaluate the sample size. We calculated the sample size based on a study on hip fracture surgery, which demonstrated that the OR value was 2.07 for the risk of POD in male patients, and the POD incidence in females was 30.2% (14). We assumed that the proportion of male and female patients was 1:1. The minimum sample size of 127 patients in the male or female group was obtained when choosing a power of 80% and a two-sided α of 0.05.

## Results

**Figure 1:**
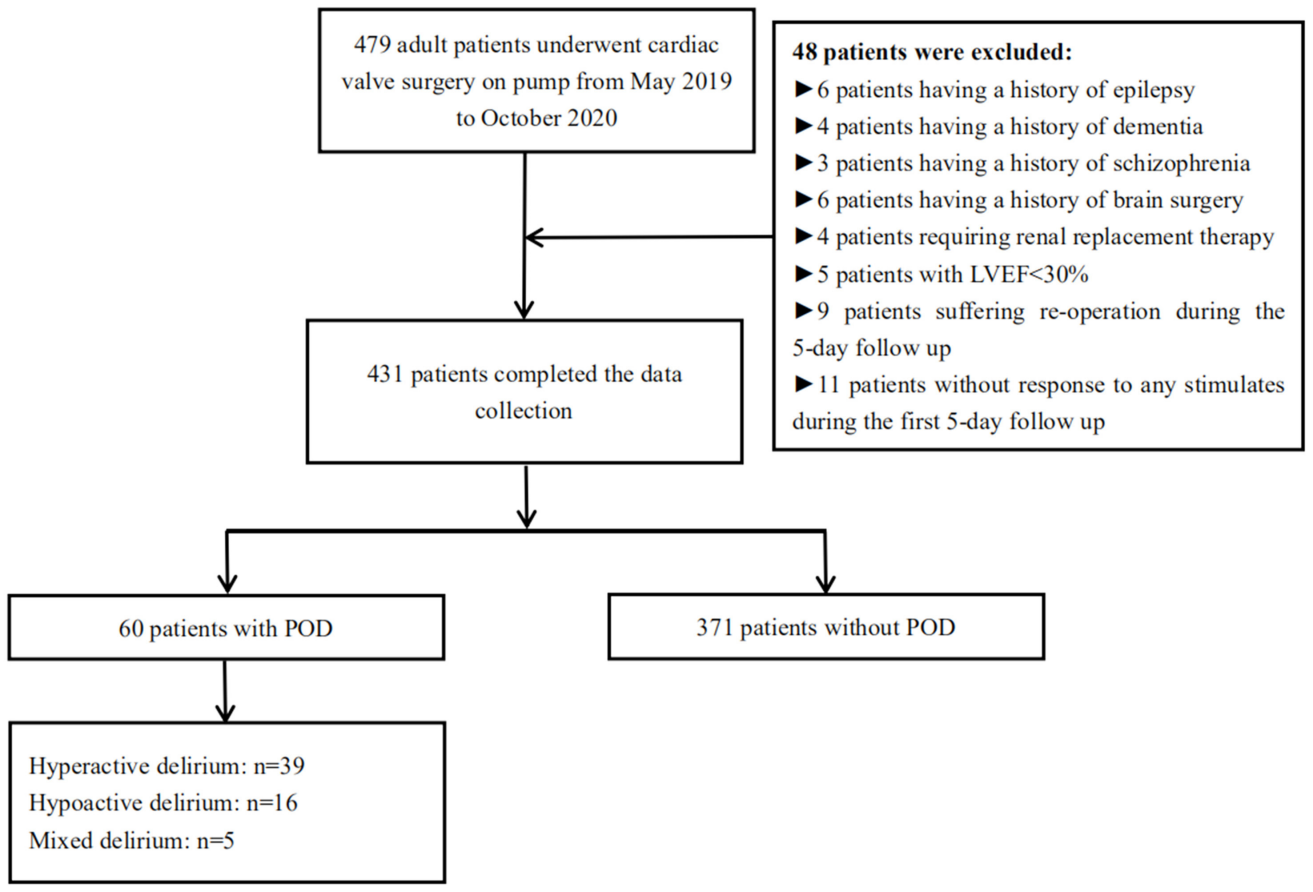
The flow chart of screening cases and grouping for this retrospective case-control study.

We identified 479 adult patients undergoing cardiac valve surgery from May 2019 to October 2020. We excluded 48 patients according to the exclusion criteria. Eventually, we collected the perioperative data in 431 enrolled patients. Sixty patients (13.9%) were diagnosed with POD (Figure 1). Thirty-nine cases (18.4%) in 212 male patients and 21 cases (9.6%) in 212 female patients developed POD.

Table 1 demonstrated the univariate analyses of demographic data or potential risk factors other than gender. The results with *P* < 0.1 included the following 11 potential risk factors: age, gender, BMI, alcohol abuse, preoperative MMSE score, preoperative sleep quality, history of cerebrovascular disease, history of DM, EuroScore, duration of mechanical ventilation, and duration of ICU stay. The collinearity diagnostics was used to judge interinfluence between the 11 independent variables, and we did not detect the severe collinearity (Supplementary Material 4). The results of unadjusted and adjusted logistic regression analyses were shown in Table 2. An unadjusted logistic regression analysis was performed to assess the relationship of gender differences and POD occurrence (unadjusted OR: 2.126, 95% CI: 1.204–3.752, *P* = 0.009). Afterward, an adjusted logistic regression was conducted by adding the risk factors with *P* < 0.1 in univariate analyses, and obtained the relationship of gender differences and POD incidence (adjusted OR: 2.213, 95% CI: 1.049–4.670, *P* = 0.037) (Table 2). The Hosmer-Lemeshow test showed good calibration of the logistic regression model (χ^2^ = 7.238, *P* = 0.511).

**Table 1:** Univariate analyses of baseline characteristics and risk factors according to POD.

	**Total**	**POD**	**No POD**	***P*-value**
	**(*n* = 431)**	**(*n* = 60)**	**(*n* = 371)**	
Age, years, median (IQR)	55 (47–62)	61 (55–68)	54 (46–61)	<0.001
Male gender, *n* (%)	212 (49.2)	39 (65.0)	173 (46.6)	0.008
BMI, kg/m^2^, median (IQR)	23.7 (21.9–26.3)	23.1 (20.4–25.6)	23.8 (22.0–26.4)	0.059
Educational level, years, median (IQR)	9 (9–12)	9 (6–12)	9 (9–12)	0.478
^a^Smoking, *n* (%)	72 (16.7)	14 (23.3)	58 (15.6)	0.138
^b^Alcohol abuse, *n* (%)	70 (16.2)	15 (25)	55 (14.8)	0.047
Preoperative MMSE score, median (IQR)	28 (26–29)	27 (26–28)	28 (26–29)	0.006
Preoperative poor sleep quality, *n* (%)	241 (55.9)	42 (70)	199 (53.6)	0.018
History of cerebrovascular disease, *n* (%)	38 (8.8)	9 (15)	29 (7.8)	0.069
History of DM, *n* (%)	23 (5.3)	6 (10)	17 (4.6)	0.083
Euroscore, median (IQR)	2 (1–4)	3 (2–4)	2 (1–4)	0.003
Duration of anesthesia, min, median (IQR)	265 (225–311)	278.5 (226.3–320)	264 (224–306)	0.384
Duration of surgery, min, median (IQR)	224 (188–269)	226.5 (182.5–285.8)	223 (188–266)	0.446
Duration of CPB, min, median (IQR)	114 (84–150)	122 (80–157.8)	112 (84–150)	0.412
Duration of aortic clamping, min, median (IQR)	84 (59–118)	91.5 (58.8–122.5)	83 (59–116)	0.434
Duration of hypothermia, min, median (IQR)	69 (47–99)	70 (45.8–103.5)	69 (47–96)	0.987
Intraoperative blood loss, ml, median, (IQR)	600 (600–600)	600 (600–600)	600 (600–600)	0.327
Intraoperative infusion volume, ml, median, (IQR)	550 (400–800)	575 (500–987.5)	500 (400–800)	0.288
Duration of mechanical ventilation, min, median, (IQR)	908 (700–1,060)	1154 (996–2,082)	875 (689–1,020)	<0.001
Duration of ICU stay, days, median, (IQR)	2 (2–4)	4 (2–5)	2 (1–4)	<0.001
The highest NRS score for pain after surgery, median, (IQR)	3 (2–4)	3 (2–4)	3 (2–4)	0.933

*POD, postoperative delirium; IQR, interquartile range; BMI, body mass index; MMSE, the Mini-Mental State Examination; DM, diabetes mellitus; CPB, cardiopulmonary bypass; ICU, intensive care unit; NRS, Numeric Rating Scale*.

a *Smoking was defined as current smoking or smoking cessation of less than 6 month*.

b *Alcohol abuse was defined as alcohol intake more than twice daily*.

**Table 2:** The logistic regression analysis of screened variables.

	**Unadjusted OR**	**95% CI for unadjusted OR**	***P-*value**	**Adjusted OR**	**95% CI for adjusted OR**	***P-*value**
Gender	2.126	1.204–3.752	0.009	2.213	1.049–4.670	0.037
Age	1.068	1.038–1.098	<0.001	1.051	1.008–1.097	0.020
BMI	0.915	0.840–0.998	0.045	0.940	0.851–1.037	0.216
Alcohol abuse	1.915	0.999–3.671	0.050	1.542	0.671–3.545	0.308
Preoperative MMSE score	0.819	0.701–0.956	0.011	1.060	0.838–1.343	0.626
Preoperative poor sleep quality	2.017	1.119–3.633	0.020	1.546	0.760–3.146	0.229
History of cerebrovascular disease	2.081	0.932–4.649	0.074	1.998	0.740–5.400	0.172
History of DM	2.314	0.874–6.126	0.091	2.208	0.697–6.995	0.178
Euroscore	1.246	1.088–1.427	0.001	0.898	0.730–1.105	0.310
Duration of mechanical ventilation	1.001	1.001–1.002	<0.001	1.001	1.001–1.002	<0.001
Duration of ICU stay	1.588	1.334–1.891	<0.001	1.310	1.082–1.586	0.006

*BMI, body mass index; CI, confidence interval; MMSE, the Mini-Mental State Examination; DM, diabetes mellitus; ICU, intensive care unit; OR, odds ratio*.

**Figure 2:**
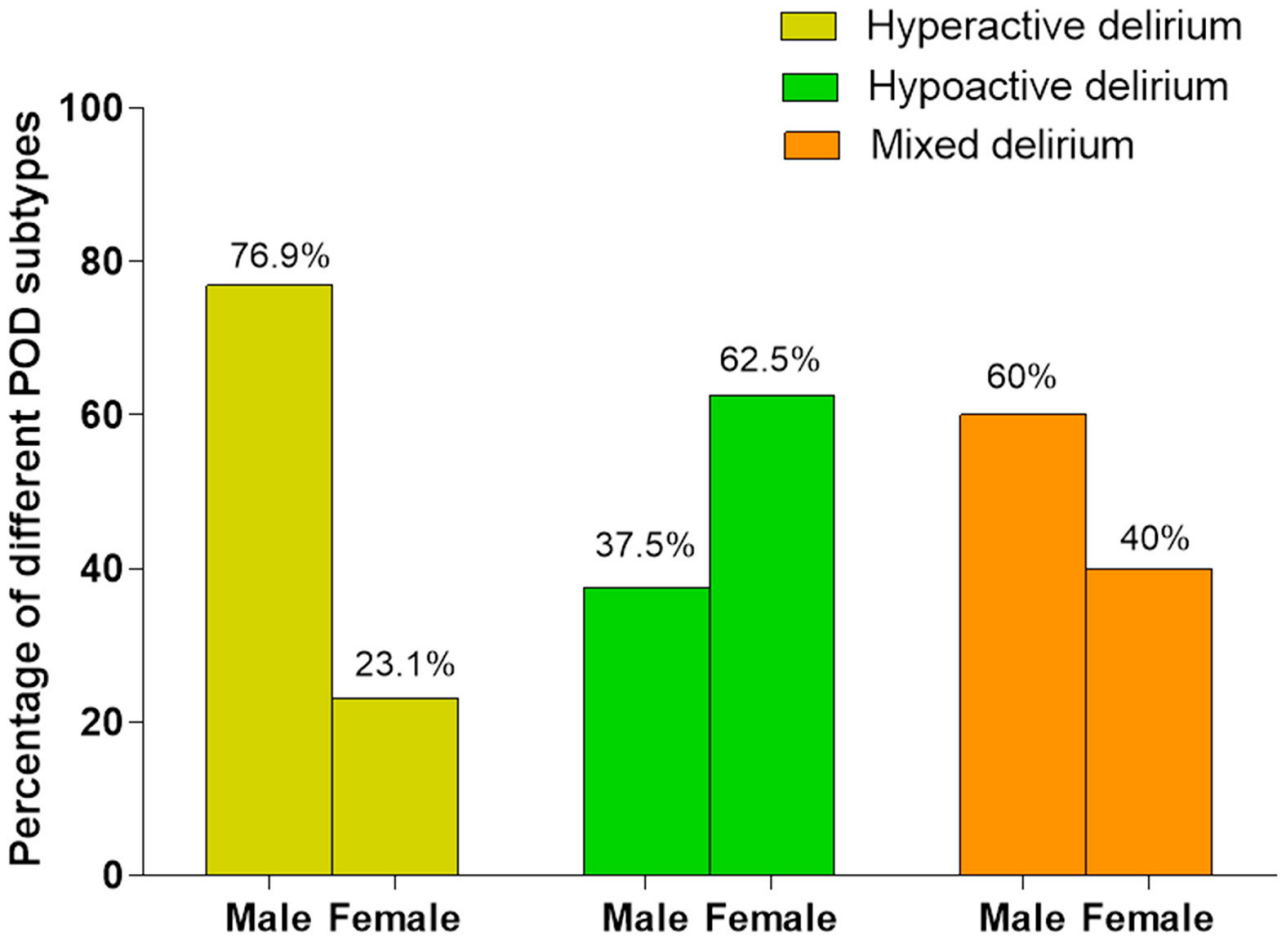
The incidence of POD subgroups in males and females.

The incidence of POD subtypes between males and females was: (1) hyperactive delirium: (males: 76.9%; females: 23.1%); (2) hypoactive delirium: (males: 37.5%; females: 62.5%); (3) mixed delirium: (males: 60%; females: 40%) (Figure 2). Considering that there were only five cases of mixed delirium, we did not conduct logistic regression analysis for this subgroup. The statistical results of relationship of gender differences and the other two delirium subtypes (hyperactive and hypoactive) were shown in Table 3: (1) hyperactive delirium: unadjusted OR: 3.846, 95% CI: 1.779–8.314, *P* = 0.001; adjusted OR: 3.384, 95% CI: 1.335–8.580, *P* = 0.010; (2) hypoactive delirium: unadjusted OR: 0.609, 95% CI: 0.217–1.706, *P* = 0.345; adjusted OR: 0.509, 95% CI: 0.147–1.766, *P* = 0.287).

**Table 3:** The logistic regression analysis of association between gender differences and occurrence of POD and its subtypes.

	**Male**	**Female**	**Unadjusted OR**	**95% CI for unadjusted OR**	***P-*value**	**Adjusted OR**	**95% CI for adjusted OR**	***P-*value**
POD (*n* = 60)	39	21	2.126	1.204–3.752	0.009	2.213	1.049–4.670	0.037
**POD subtypes**								
Hyperactive delirium (*n* = 39)	30	9	3.846	1.779–8.314	0.001	3.384	1.335–8.580	0.010
Hypoactive delirium (*n* = 16)	6	10	0.609	0.217–1.706	0.345	0.509	0.147–1.766	0.287
Mixed delirium (*n* = 5)	3	2	-	-	-	-	-	-

*CI, confidence interval; POD, postoperative delirium; OR, odds ratio*.

A stratification analysis was performed based on age (<60 and ≥60 years old). The statistical results of association between gender differences and POD were shown in Table 4. The number of patients aged younger than 60 years was 285 (male: 134; female: 151), and the number of patients with POD was 18 and 7 in males and females, respectively. The number of patients aged 60 years or older was 146 (male: 78; female: 68), and 21 in males and 14 in females developed POD. The association between male gender and POD occurrence was: (1) aged <60 years: OR: 3.192, 95% CI: 1.289 to 7.904, *P* = 0.012; adjusted OR: 4.384, 95% CI, 1.318 to 14.586, *P* = 0.016; (2) aged ≥60 years: OR: 1.421, 95% CI: 0.657 to 3.075, *P* = 0.372; adjusted OR: 1.798, 95% CI: 0.661 to 4.895, *P* = 0.251).

**Table 4:** Stratification analysis by age.

**Age**	**Number of Males with POD**	**Number of Females with POD**	**Unadjusted OR**	**95% CI for Unadjusted OR**	***P*-value**	**Adjusted OR**	**95% CI for adjusted OR**	***P*-value**
<60 years	18/134	7/151	3.192	1.289–7.904	0.012	4.384	1.318–14.586	0.016
(*n* = 285)								
≥60 years	21/78	14/68	1.421	0.657–3.075	0.372	1.798	0.661–4.895	0.251
(*n* = 146)								

*CI, confidence interval; POD, postoperative delirium; OR, odds ratio*.

## Discussion

This study discovered that male gender was a strong risk factor in POD occurrence in patients undergoing open heart cardiac valve surgery. Furthermore, the hyperactive delirium was more common in males. Besides, the males demonstrated the higher incidence of POD in patients aged younger than 60 years old.

Epidemiological studies demonstrated that the males may experience more risk factors intimately associated with impairment in cognitive-related brain domains, like obstructive sleep apnea, alcohol dependence, psychological stress for disease, and so on, which may be the main causes of POD (25–29). However, the specific mechanism of gender associated with POD occurrence is still unclear.

Male gender may be an important risk factor in neuropsychiatric problems in humans and animals, meanwhile, estrogen may be a protective factor in individuals with potential cognitive impairment. According to an epidemiological investigation, of the patients with an implantable cardioverter defibrillator, the males were more likely to develop anxiety symptoms (30). An experiment from a rat model of coronary artery ligation exhibited a higher plasma concentration of depression/anxiety-related neutrophil gelatinase associated lipocalin (NGAL) in the male rats, and presented stronger signs of depressive-like behavior and cognitive dysfunction in males (31). The higher level of preoperative NGAL in patients with critical illness might be a vital mechanism of delirium occurrence in males (32). Another animal study showed that estrogen synthesis played a positive role of neuropsychiatric effects in adult rats, because high estrogen levels could elevate noradrenaline and the dopaminergic turnover rates in neuropsychiatry associated brain domains, like hippocampus and prefrontal cortex (PFC) (33). Also, estradiol could significantly improve the spatial working memory in gonadectomized rats (34). Preoperative cognitive dysfunction played an important role in delirium occurrence in cardiac surgery patients (35).

The males could manifest more pronounced neuropsychiatric disorders following acute stress compared with females. Cardiac surgery is actually an acute stress factor and produces complicated neuroendocrine changes and neuroinflammatory response, and eventually may lead to cognitive and/or mental disorders (36–38). In the corticolimbic brain domains, endocrine and behavioral responses to stress is activated through corticotropin-releasing factor (CRF) signaling. CRF binding protein (CRFBP) also plays an important role in stress responses, since it can enhance the affinity of CRF and its receptor 1 (CRF1). Sex differences in CRF signaling changes following acute stress may produce different outcomes of neuropsychiatric disorders (39). Besides, relative to female rats, the males with chronic stress demonstrated striking reduction in acute stress-induced c-Fos expression in the medial PFC, hippocampus, and paraventricular nucleus of the hypothalamus (40). These changes may also be an important mechanism in higher incidence of POD in males.

Previous studies exhibited that the incidence of hypoactive delirium more commonly occurred in ICU patients following cardiac surgery, which was associated with mechanical ventilation and sedative administration (20, 41). In this study, we selected the patients with isolated cardiac valve surgery, and most of them were stopped sedative infusion and extubated within the first postoperative 24 h. The delirium assessment for the first time was conducted within the first postoperative 24 h, and the specific time of assessment was as close as possible to 24 h of the end of the surgery to minimize the impact of anesthetics on the consciousness of patients. These reasons may be related to the higher incidence of hyperactive delirium in this study. Furthermore, some studies have identified that male gender is a risk factor of hyperactive delirium in post-surgery patients, which was consistent with our results (42, 43). However, the concrete mechansim is unclear, and requires further investigation. Additionally, although older age was an identified risk factor related to POD occurrence in cardiac surgery patients, we obtained an interesting result that the significant difference in POD occurrence between males and females was in patients aged younger than 60 years old. Also, this result need to be further proved through a prospective study with larger sample size.

In this study, we obtained 11 possible risk factors of POD through univariate logistic regression analysis. Given that we might acquire misleading statistical results generated from these covariates due to severe multicollinearity, we performed multicollinearity analysis to detect the overlapping risk factors on the outcome (24, 44). Eventually, we did not find the severe collinearity among these variables. We analyzed the relationship of gender differences and delirium subtypes, and found that both of unadjusted and adjusted OR values were consistent with *P*-values in delirium and hyperactive subtype, which meant that these results were robust. In the logistic regression model, the OR values were commonly used to accurately represent the statistical association between risk factors and the results rather than the *P*-values (45). However, the study with a small sample size may derive bias of OR values, 95% CI containing 1, and result in poor credibility of the results (46, 47). Therefore, a prospective cohort study with larger sample size is required to further prove the correlation of gender differences and occurrence of hypoactive or mixed delirium.

There are several limitations in this study. First, it is a retrospective case-control study, the bias of data collection are inevitable, and it may affect the outcomes. However, in this current study, there was no data loss in the included patients, and two researchers independently collected data, and another researcher reviewed the data. We thereby reduced the effect of bias of data collection on the results as far as we can. Second, we estimated the sample size according to the study on non-cardiac surgery, which might not provide the relatively accurate OR values and percentages of POD in exposure and control groups in cardiac surgery patients. According to this study, the adjusted OR is 2.213, and the incidence of POD in the females is about 10%, and we yield a sample size of 209 patients in each group when we select a power of 80% and a two-sided α of 0.05. Therefore, the sample size in this study is sufficient to acquire a robust result. However, the sample size of delirium subtypes may be insufficient, and these results are required to be proved based on the study with larger sample size. Third, the endpoint and some variables of this study were acquired only by the subjective measurements, which might lead to measurement bias, although these subjective methods were reliable according to previous studies. We do believe that the results can be more credible if the objective methods are added. This subjective study will provide a direction for the further study by the objective methods in the future.

## Conclusion

The male gender is a significant risk factor in POD occurrence in patients following cardiac valve surgery on pump. Moreover, the hyperactive delirium occurs more in male patients. Besides, the males demonstrated the higher incidence of POD in patients aged younger than 60 years old. The anesthetists, surgeons, and doctors in ICU should pay more attention on the male patients, and take active preventive measures when necessary. Given the limitations of retrospective study, in the future, a high-quality prospective cohort study with large sample size will be required to further prove these results.

## Data Availability Statement

The original contributions presented in the study are included in the article/Supplementary Material, further inquiries can be directed to the corresponding author.

## Ethics Statement

The studies involving human participants were reviewed and approved by the Ethics Committee of the Chinese Academy of Medical Sciences Fuwai Hospital. The patients/participants provided their written informed consent to participate in this study. Written informed consent was obtained from the individual(s) for the publication of any potentially identifiable images or data included in this article.

## Author Contributions

HW and XG helped with the conception, data collection, and drafting the manuscript. XZ was responsible for data collection. YL reviewed the data. ZZ and YJ conducted the statistical analysis and reviewed the manuscript. SY and FY designed the study and supervised the entire process. All authors helped with the final approval of the version to be published.

## Conflict of Interest

The authors declare that the research was conducted in the absence of any commercial or financial relationships that could be construed as a potential conflict of interest.

## Publisher's Note

All claims expressed in this article are solely those of the authors and do not necessarily represent those of their affiliated organizations, or those of the publisher, the editors and the reviewers. Any product that may be evaluated in this article, or claim that may be made by its manufacturer, is not guaranteed or endorsed by the publisher.

## Supplementary Material

Supplementary Material 1 Pittsburgh sleep quality index (PSQI).

Supplementary Material 2 The confusion assessment method for the intensive care unit (CAM-ICU).

Supplementary Material 3 The Richmond agitation-sedation scale (RASS).

Supplementary Material 4 The collinearity diagnostics of the potential risk factors.
