# Supplementary material for: Gender Differences and Postoperative Delirium in Adult Patients Undergoing Cardiac Valve Surgery
Source: Front Cardiovasc Med. 2021 Nov 23;8:751421. doi: 10.3389/fcvm.2021.751421 (PMC8649844; doi:10.3389/fcvm.2021.751421)
Supplement: Supplementary Material 1 — Pittsburgh sleep quality index (PSQI). [file Table_1.DOCX]

Pittsburgh Sleep Quality Index (PSQI)

| Name: ID #: Date: Age: |
| --- |
| Instructions |
| The following questions relate to your usual sleep habits during the past month only. Your answers should indicate the most accurate reply for the majority of days and nights in the past month. |
| Please answer all questions |
| 1. During the past month, when have you usually gone to bed at night?--Usual bed time:____ |
| 2.During the past month, how long (in minutes) has it usually taken you to fall asleep each night?---Number of minutes:___________ |
| 3. During the past month, when have you usually gotten up in the morning?--- Usual gotting up time:_____________ |
| 4. During the past month, how many hours of actual sleep did you get at night? (This may be different than the number of hours you spend in bed.)--- Hours of sleep per night:____________ |
| For each of the remaining questions, check the one best response. Please answer all questions. |
| 5. During the past month, how often have you had trouble sleeping because you... |
| (a) Cannot get to sleep within 30 minutes.  Not during the past month___; Less than once a week___; Once or twice a week___; Three or more times a week___. |
| (b) Wake up in the middle of the night or early morning.  Not during the past month___; Less than once a week___; Once or twice a week___; Three or more times a week___. |
| (c) Have to get up to use the bathroom.  Not during the past month___; Less than once a week___; Once or twice a week___;Three or more times a week___. |

Continued

| (d) Cannot breathe comfortably.  Not during the past month___; Less than once a week___; Once or twice a week___; Three or more times a week___.  (e) Cough or snore loudly.  Not during the past month___; Less than once a week___; Once or twice a week___;Three or more times a week___. |
| --- |
| (f) Feel too cold.  Not during the past month___; Less than once a week___; Once or twice a week___;Three or more times a week___. |
| (g) Feel too hot.  Not during the past month___; Less than once a week___; Once or twice a week_ __; Three or more times a week___. |
| (h) Had bad dreams.  Not during the past month___; Less than once a week___; Once or twice a week_ __; Three or more times a week___. |
| (i) Have pain.  Not during the past month___; Less than once a week___; Once or twice a week___; Three or more times a week___. |
| (j) Other reason(s), please describe_____________________________________________.  How often during the past month have you had trouble sleeping because of this?  Not during the past month___; Less than once a week___; Once or twice a week_ __; Three or more times a week___. |
| 6. During the past month, how would you rate your sleep quality overall?  Very good____; Fairly good____; Fairly bad____; Very bad____. |
| 7. During the past month, how often have you taken medicine (prescribed or “over the  counter”) to help you sleep? |

Continued

| Not during the past month___; Less than once a week___; Once or twice a week___;Three or more times a week___.  8. During the past month, how often have you had trouble staying awake while driving, eating meals, or engaging in social activity?  Not during the past month___; Less than once a week___; Once or twice a week___;Three or more times a week___. |
| --- |
| 9. During the past month, how much of a problem has it been for you to keep up enough enthusiasm to get things done?  No problem at all____; Only a very slight problem___; Somewhat of a problem___; A very big problem___. |
| 10. Do you have a bed partner or roommate?  No bed partner or roommate___; Partner/roommate in other room___; Partner in same room, but not same bed___; Partner in same bed___. |
| If you have a roommate or bed partner, ask him/her how often in the past month you have had... |
| (a) Loud snoring.  Not during the past month___; Less than once a week___; Once or twice a week___; Three or more times a week___. |
| (b) Long pauses between breaths while asleep.  Not during the past month___; Less than once a week___; Once or twice a week___; Three or more times a week___. |
| (c) Legs twitching or jerking while you sleep.  Not during the past month___; Less than once a week___; Once or twice a week___; Three or more times a week___. |
| (d) Episodes of disorientation or confusion during sleep.  Not during the past month___; Less than once a week___; Once or twice a week___; Three or more times a week___. |

Continued

| (e) Other restlessness while you sleep: please describe_______________________________.  Not during the past month___; Less than once a week___; Once or twice a week___; Three or more times a week___. |
| --- |

Scoring instructions for the Pittsburgh Sleep Quality Index

| Component 1: Subjective sleep quality |
| --- |
| Examine question #6, and assign scores as follows:  " Very good" score 0; "Fairly good" score 1; "Fairly bad" score 2; "Very bad score" 3 |
| Component 1 score:_______ |
| Component 2: Sleep latency |
| 1. Examine question #2, and assign scores as follows:  "≤15 minutes" score 0; "16-30 minutes" score 1; "31-60 minutes" score 2; ">60 minutes" score 3  Question #2 score:_______ |
| 2. Examine question #5a, and assign scores as follows:  "Not during the past month" score 0; "Less than once a week" score 1; "Once or twice a week" score 2; "Three or more times a week" score 3  Question #5 score:_______ |
| 3. Add #2 score and #5a score Sum of #2 and #5a:_______ |
| 4. Assign component 2 score as follows:  Sum of #2 and #5a score "0" component 2 score "0"; Sum of #2 and #5a score "1-2" component 2 score "1"; Sum of #2 and #5a score "3-4" component 2 score "2"; Sum of #2 and #5a score "5-6" component 2 score "3" |
| Component 2 score:_______ |
| Component 3: Sleep duration |
| Examine question #4, and assign scores as follows:  "> 7 hours" score 0; "6-7 hours" score 1; "5-6 hours" score 2; "< 5 hours" score 3 |
| Component 3 score:_______ |
| Component 4: Habitual sleep efficiency.  (1) Write the number of hours slept (question # 4) here:________. |

Continued

| (2) Calculate the number of hours spent in bed:____________.  Getting up time (question #3):__________.  Bedtime (question #1):_________.  Number of hours spent in bed:________.  (3) Calculate habitual sleep efficiency as follows:  (Number of hours slept/Number of hours spent in bed) × 100 = Habitual sleep efficiency (%)  (4) Assign component 4 score as follows:  "> 85%" score 0; "75-84%" score 1; "65-74%" score 2; "< 65%" score 3 |
| --- |
| Component 4 score:_______ |
| Component 5: Sleep disturbances |
| (1) Examine questions # 5b-5j, and assign scores for each question as follows:  "Not during the past month" score 0; "Less than once a week" score 1; "Once or twice a week" score 2; "Three or more times a week" score 3  (2) Add the scores for questions # 5b-5j:_______.  (3) Assign component 5 score as follows:  "0" score 0; "1-9" score 1; "10-18" score 2; "19-27" score 3 |
| Component 5 score:_______ |
| Component 6: Use of sleeping medication |
| Examine question # 7 and assign scores as follows:  "Not during the past month" score 0; "Less than once a week" score 1; "Once or twice a week" score 2; "Three or more times a week" score 3. |
| Component 6 score:_______ |
| Component 7: Daytime dystunction |
| (1) Examine question # 8, and assign scores as follows:  "Never" score 0; "Once or twice a week" score 1; "Once or twice a week" score 2;  "Three or more times a week" score 3. Question #8 score:_______ |

Continued

| (2) Examine question # 9, and assign scores as follows:  "No problem at all" score 0; "Only a very slight problem" score 1; "Somewhat a problem" score 2; "A very big problem" score 3. Question #9 score:_______  (3) Add the scores for question # 8 and # 9: Sum of #8 and #9:______  (4) Assign component 7 score as follows:  "0" score 0; "1-2" score 1; "3-4" score 2; "5-6" score 3 |
| --- |
| Component 7 score:_______ |
| Global PSQI Score |
| Add the seven component scores together: |
| Global PSQI Score:_______ |
