# Supplementary material for: Gender Differences and Postoperative Delirium in Adult Patients Undergoing Cardiac Valve Surgery
Source: Front Cardiovasc Med. 2021 Nov 23;8:751421. doi: 10.3389/fcvm.2021.751421 (PMC8649844; doi:10.3389/fcvm.2021.751421)
Supplement: Supplementary Material 2 — The confusion assessment method for the intensive care unit (CAM-ICU). [file Table_2.DOCX]

Suppl. table 2

| Feature 1: Acute Onset and Fluctuating Course | Score | Check here if present |
| --- | --- | --- |
| Is the patient different than his/her baseline mental status?  OR  Has the patient had any fluctuation in mental status in the past 24 hours as evidenced by fluctuation on a sedation/level of consciousness scale (i.e.,RASS/SAS), GCS, or previous delirium assessment? | Either  question  Yes |  |
| Feature 2: Inattention |  |  |
| Letters Attention Test (See training manual for alternate Pictures)  Directions: Say to the patient, “I am going to read you a series of 10 letters. Whenever you hear the letter ‘A,’ indicate by squeezing my hand.” Read letters from the following letter list in a normal tone 3 seconds apart. S A V E A H A A R T or C A S A B L A N C A or A B A D B A D A A Y  Errors are counted when patient fails to squeeze on the letter “A” and when the patient squeezes on any letter other than “A.” | Number  of  Errors >2 |  |
| Feature 3: Altered Level of Consciousness |  |  |
| Present if the Actual RASS score is anything other than alert and calm (zero) | RASS  anything  other  than zero |  |
| Feature 4:Disorganized Thinking |  |  |
| Yes/No Questions (Use group A or group B to test, if necessary, group A and B can be used interchangeably)  A:  1. Will a stone float on water?  2. Are there fish in the sea?  3. Does one pound weigh more than two pounds?  4. Can you use a hammer to pound a nail?  B: 1. Will a leaf float on water?  2. Are there elephants in the sea?  3. Does two pounds weigh more than one pound?  4. Can you use a hammer to saw wood?  Errors are counted when the patient incorrectly answers a question.  Command  Say to patient: “Hold up this many fingers” (Hold 2 fingers in front of patient) “Now do the same thing with the other hand” (Do not repeat number of fingers) | Combined  number  of  errors >1 |  |

Continued

| *If the patient is unable to move both arms, for 2nd part of  command ask patient to “Add one more finger”  An error is counted if patient is unable to complete the entire command. |  |  |
| --- | --- | --- |
| Delirium is diagnosed when a patient meets Feature 1 plus 2 and either 3 or 4. | | |
