# Supplementary material for: Gender Differences and Postoperative Delirium in Adult Patients Undergoing Cardiac Valve Surgery
Source: Front Cardiovasc Med. 2021 Nov 23;8:751421. doi: 10.3389/fcvm.2021.751421 (PMC8649844; doi:10.3389/fcvm.2021.751421)
Supplement: Supplementary Material 3 — The Richmond agitation-sedation scale (RASS). [file Table_3.DOCX]

Suppl. table 3

| Score | Term | Description |
| --- | --- | --- |
| +4 | Combative | Overtly combative or violent; immediate danger to staff |
| +3 | Very agitated | Pulls on or removes tube(s) or catheter(s) or has aggressive behavior toward staff |
| +2 | Agitated | Frequent nonpurposeful movement or patient-ventilator dyssynchrony |
| +1 | Restless | Anxious or apprehensive but movements not aggressive or vigorous |
| 0 | Alert and calm |  |
| -1 | Drowsy | Not fully alert, but has sustained (more than 10 s) awakening, with eye contact, to voice |
| -2 | Light sedation | Briefly (less than 10 s) awakens with eye contact to voice |
| -3 | Moderate sedation | Any movement (but no eye contact) to voice |
| -4 | Deep sedation | No response to voice, but any movement to physical stimulation |
| -5 | Unarousable | No response to voice or physical stimulation |
