# Supplementary material for: Gender Differences and Postoperative Delirium in Adult Patients Undergoing Cardiac Valve Surgery
Source: Front Cardiovasc Med. 2021 Nov 23;8:751421. doi: 10.3389/fcvm.2021.751421 (PMC8649844; doi:10.3389/fcvm.2021.751421)
Supplement: Supplementary Material 4 — The collinearity diagnostics of the potential risk factors. [file Table_4.DOC]

系数a	
模型	未标准化系数	标准化系数	t	显著性	共线性统计	
	B	标准错误	Beta			容差	
1	(常量)	-.208	.393		-.529	.597		
	性别	.072	.034	.104	2.084	.038	.762	
	年龄	.003	.002	.103	1.694	.091	.514	
	BMI	-.007	.005	-.071	-1.589	.113	.951	
	饮酒	.045	.045	.048	1.005	.315	.815	
	术前MMSE	.003	.011	.016	.281	.779	.579	
	术前睡眠质量	.036	.033	.052	1.107	.269	.843	
	术前脑梗史	.080	.057	.066	1.415	.158	.870	
	术前糖尿病史	.107	.068	.070	1.580	.115	.973	
	EuroScore	-.004	.010	-.022	-.396	.692	.617	
	术后带管时间	.000	.000	.281	5.547	.000	.735	
	ICU停留时间	.028	.010	.139	2.698	.007	.712	

系数a	
模型	共线性统计	
	VIF	
1	(常量)		
	性别	1.313	
	年龄	1.947	
	BMI	1.051	
	饮酒	1.227	
	术前MMSE	1.727	
	术前睡眠质量	1.186	
	术前脑梗史	1.150	
	术前糖尿病史	1.028	
	EuroScore	1.621	
	术后带管时间	1.360	
	ICU停留时间	1.404	

a. 因变量：术后谵妄	
